# Food consumption rated by quality index diet (IQD) in pregnant women with gestational diabetes mellitus

**DOI:** 10.1186/1758-5996-7-S1-A67

**Published:** 2015-11-11

**Authors:** Lilian Barros de Sousa Moreira Reis, Iracema de Mattos Paranhos Calderon, Cláudia Vicari Bolognani, Adriano Dias, Adriano Dias

**Affiliations:** 1 Secretaria de Saúde do Distrito Federa/FMB-UNESP, Asa Sul, Brazil

## Background

Proper nutrition is important during pregnancy, and especially in those complicated by diabetes.

## Objective

Assess food intake by diet quality index (IQD) in pregnant women with Melitos Gestational Diabetes (GDM).

## Materials and methods

Cohort study transversal and descriptive in 65 pregnant women with GDM. Dietary intake was determined by 24-hour dietary recall (24HR) and a Food Frequency Questionnaire (FFQ), and qualified IQD.

## Results and discussion

67.7% of the women had body mass index (BMI) before pregnancy ≥ 25 kg/m 2. The caloric value was observed in 24HR 1657±532 kcal. According to the IQD, the diet was adequate in 51.6% of pregnant women. The worst score components were vegetables and dairy products. The intake of meat, sodium and total fat received the highest scores.

## Conclusion

IQD, referenced by 24HR and applied as a tool for nutritional assessment showed that the diet was considered inadequate or in need of adjustment in half of the pregnant population evaluated. These inadequacies were related to low intake of vegetables and milk and milk products. These Results indicate the need to prioritize educational prenatal, to encourage the consumption of vegetables and dairy products among these pregnant women with GDM.

